# Supplementary material for: A geometric morphometric protocol to correct postmortem body arching in fossil fishes
Source: PeerJ. 2024 May 31;12:e17436. doi: 10.7717/peerj.17436 (PMC11146327; doi:10.7717/peerj.17436)

TABLE 1. Description of the landmarks and semilandmarks used in the digitization of the fossil fishes.

| Number | Type | Descriptions |
| --- | --- | --- |
| 1 | Landmark | Anterior point of the dorsal fin base (dorsal extreme of first pterygiophore) |
| 2 | Landmark | Posterior point of the dorsal fin base (dorsal extreme of last pterygiophore) |
| 3 | Landmark | Dorsal point of the anterior extreme of the caudal peduncle |
| 4 | Landmark | Middle point of the anterior extreme of the caudal peduncle (articulation between the last vertebra and the hypural) |
| 5 | Landmark | Ventral point of the anterior extreme of the caudal peduncle |
| 6 | Landmark | Posterior point of the anal fin base (ventral extreme of last pterygiophore) |
| 7 | Landmark | Anterior point of the anal fin base (ventral extreme of first pterygiophore) |
| 8 | Landmark | Anterior point of the pelvic bone |
| 9 | Landmark | Articulation point of the pectoral fin with the pectoral girdle |
| 10 | Landmark | Antero-ventral point of the pectoral girdle |
| 11 | Landmark | Postero-ventral point of the lower jaw |
| 12 | Landmark | Anterior point of the premaxilla |
| 13 | Landmark | Dorsalmost point of the cranium |
| 14 | Landmark | Postero-dorsal point of the skull roof |
| 15 | Landmark | Crossing point of the vertebral column with the posterior margin of the cranium (opercle) |
| 16-19 | Semilandmark | Semilandmarks placed equidistantly along the vertebral column between landmarks 4 and 15 |

TABLE 2. Descriptive values of the IC (index of curvature) of the original sample.

| Min. | 1st Qu. | Median | Mean | 3rd Qu. | Max. |
| --- | --- | --- | --- | --- | --- |
| 0.000531 | 0.005275 | 0.010683 | 0.013683 | 0.019303 | 0.064325 |

TABLE 3. Multivariate regression of the Procrustes Data to the IC of the original sample.

|  | Df | SS | MS | Rsq | F | Z | Pr(>F) |
| --- | --- | --- | --- | --- | --- | --- | --- |
| Index of curvature | 1 | 0.11640 | 0.116396 | **0.18047** | 20.26 | 5.6062 | **0.001 **** |
| Residuals | 92 | 0.52856 | 0.005745 | 0.81953 |  |  |  |
| Total | 93 | 0.64496 |  |  |  |  |  |

TABLE 4. Regression of the first PC to the IC of the original sample.

|  |  | Estimate | Std. Error | t value | Pr(>\|t\|) |
| --- | --- | --- | --- | --- | --- |
| PC1 | Index of curvature | -0.5245 | 0.4216 | 1.244 | 0.217 |
| PC2 | Index of curvature | 2.9576 | 0.2109 | 14.03 | **< 2e-16 ***** |
| PC3 | Index of curvature | 0.1510 | 0.2734 | 0.552 | 0.582 |
| PC4 | Index of curvature | -0.04482 | 0.24806 | -0.181 | 0.857 |
| PC5 | Index of curvature | 0.3318 | 0.1579 | 2.101 | **0.0383** |
| PC6 | Index of curvature | 0.2622 | 0.1346 | 1.948 | 0.0545 |

TABLE 5. Linear regression of the IC to the Centroid size (centroid size is calculated as the square root of the sum of the squared distances between the landmarks).

|  | Estimate | Std. Error | t value | Pr(>\|t\|) |
| --- | --- | --- | --- | --- |
| Intercept | 3.0258 | 0.5289 | 5.721 | 1.31e-07*** |
| Centroid size | -2.0325 | 0.5271 | -3.856 | **0.000213***** |

TABLE 6. Differences between the IC in *Rubiesichthys gregalis* and *Gordichthys conquensis.*Welch’s t-test results.

| t | df | p-value |
| --- | --- | --- |
| 0.48348 | 57 | 0.6306 |

TABLE 7. Mean Procrustes total variances for each method

| Original data | Regression-unbending data | Tps-unbending data |
| --- | --- | --- |
| 0.006860564 | 0.005578429 | 0.005566089 |

TABLE 8. Pairwise test of total mean Procrustes variance between methods.

|  | Original data | Regression-unbending data | Tps-unbending data |
| --- | --- | --- | --- |
| Original data | 1.000 | 0.022 | 0.020 |
| Regression- unbending data |  | 1.000 | 0.978 |
| Tps- unbending data |  |  | 1.000 |

TABLE 9. Total mean Procrustes variance per landmark. Landmarks follow the order indicated in Figure 1C,D and Table 1.

| Landmarks | Original data | Regression- unbending data | Tps- unbending data | Original data- Regression data | Original data- Tps data |
| --- | --- | --- | --- | --- | --- |
| 1 | 0.00107 | 0.000910 | 0.000840 | 0.00016 | 0.00023 |
| 2 | 0.000796 | 0.000706 | 0.000683 | 0.00009 | 0.000113 |
| 3 | 0.000389 | 0.000219 | 0.000294 | 0.00017 | 0.000095 |
| 4 | 0.000251 | 0.000135 | 0.000133 | 0.000116 | 0.000118 |
| 5 | 0.000237 | 0.000169 | 0.000170 | 0.000068 | 0.000067 |
| 6 | 0.000282 | 0.000265 | 0.000269 | 0.000017 | 0.000013 |
| 7 | 0.000437 | 0.000384 | 0.000399 | 0.000053 | 0.000038 |
| 8 | 0.000939 | 0.000769 | 0.000863 | 0.00017 | 7.6E-05 |
| 9 | 0.000388 | 0.000335 | 0.000370 | 0.000053 | 0.000018 |
| 10 | 0.000400 | 0.000352 | 0.000426 | 0.000048 | -0.000026 |
| 11 | 0.000367 | 0.000273 | 0.000243 | 0.000094 | 0.000124 |
| 12 | 0.000530 | 0.000355 | 0.000227 | 0.000175 | 0.000303 |
| 13 | 0.000315 | 0.000268 | 0.000295 | 0.000047 | 0.00002 |
| 14 | 0.000361 | 0.000339 | 0.000299 | 0.000022 | 0.000062 |
| 15 | 0.000170 | 0.000160 | 0.000101 | 0.00001 | 0.000069 |

Figure 1. PCA plots from the original, regression-unbending method and tps-unbending method data, showing PC1 and PC2. *Rubiesichthys gregalis* is represented by purple squares and *Gordichthys conquensis* by orange circles.


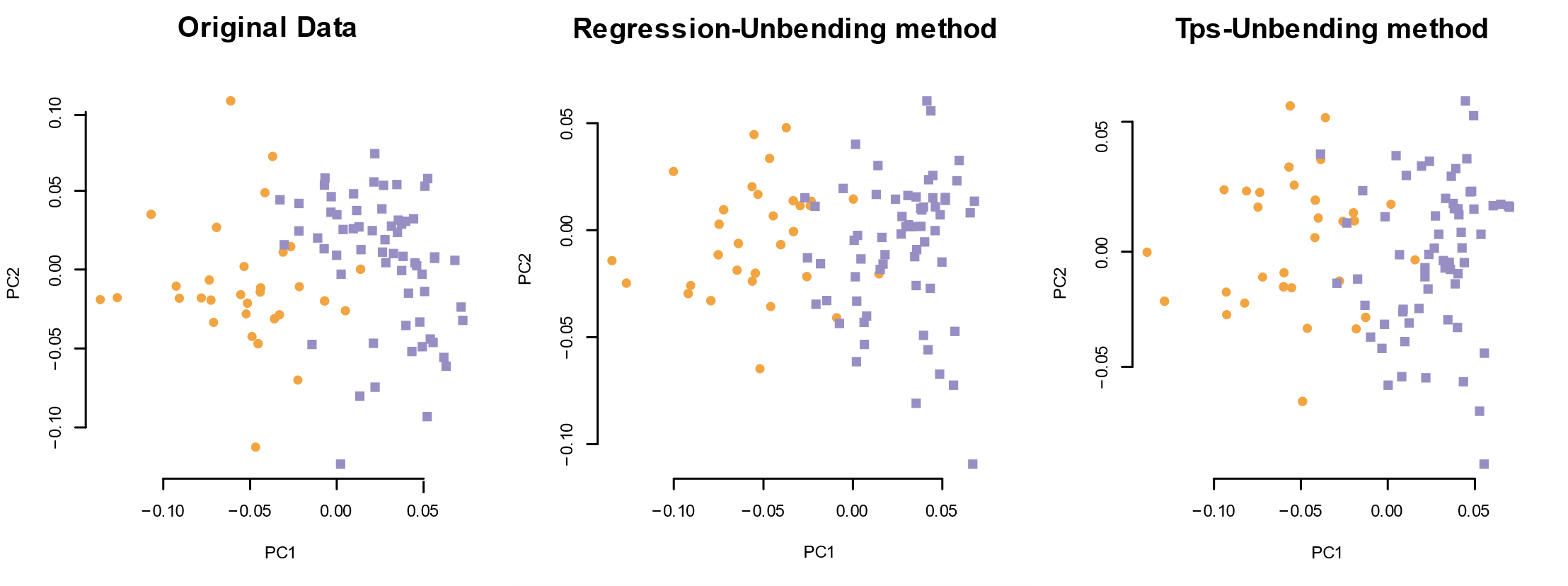

Supplement: Supplemental Information 3 [file peerj-12-17436-s003.docx]
